# Investigating the Impact of Prompt Engineering on the Performance of Large Language Models for Standardizing Obstetric Diagnosis Text: Comparative Study

**DOI:** 10.2196/53216

**Published:** 2024-02-08

**Authors:** Lei Wang, Wenshuai Bi, Suling Zhao, Yinyao Ma, Longting Lv, Chenwei Meng, Jingru Fu, Hanlin Lv

**Affiliations:** 1 BGI Research Shenzhen China; 2 The People's Hospital of Guangxi Zhuang Autonomous Region Guangxi China

**Keywords:** obstetric data, similarity embedding, term standardization, large language models, LLMs

## Abstract

**Background:**

The accumulation of vast electronic medical records (EMRs) through medical informatization creates significant research value, particularly in obstetrics. Diagnostic standardization across different health care institutions and regions is vital for medical data analysis. Large language models (LLMs) have been extensively used for various medical tasks. Prompt engineering is key to use LLMs effectively.

**Objective:**

This study aims to evaluate and compare the performance of LLMs with various prompt engineering techniques on the task of standardizing obstetric diagnostic terminology using real-world obstetric data.

**Methods:**

The paper describes a 4-step approach used for mapping diagnoses in electronic medical records to the International Classification of Diseases, 10th revision, observation domain. First, similarity measures were used for mapping the diagnoses. Second, candidate mapping terms were collected based on similarity scores above a threshold, to be used as the training data set. For generating optimal mapping terms, we used two LLMs (ChatGLM2 and Qwen-14B-Chat [QWEN]) for zero-shot learning in step 3. Finally, a performance comparison was conducted by using 3 pretrained bidirectional encoder representations from transformers (BERTs), including BERT, whole word masking BERT, and momentum contrastive learning with BERT (MC-BERT), for unsupervised optimal mapping term generation in the fourth step.

**Results:**

LLMs and BERT demonstrated comparable performance at their respective optimal levels. LLMs showed clear advantages in terms of performance and efficiency in unsupervised settings. Interestingly, the performance of the LLMs varied significantly across different prompt engineering setups. For instance, when applying the self-consistency approach in QWEN, the *F*_1_-score improved by 5%, with precision increasing by 7.9%, outperforming the zero-shot method. Likewise, ChatGLM2 delivered similar rates of accurately generated responses. During the analysis, the BERT series served as a comparative model with comparable results. Among the 3 models, MC-BERT demonstrated the highest level of performance. However, the differences among the versions of BERT in this study were relatively insignificant.

**Conclusions:**

After applying LLMs to standardize diagnoses and designing 4 different prompts, we compared the results to those generated by the BERT model. Our findings indicate that QWEN prompts largely outperformed the other prompts, with precision comparable to that of the BERT model. These results demonstrate the potential of unsupervised approaches in improving the efficiency of aligning diagnostic terms in daily research and uncovering hidden information values in patient data.

## Introduction

The advancement of medical informatization has resulted in the accumulation of vast amounts of electronic medical records (EMRs) in hospitals, giving rise to medical big data [1]. These data hold significant research value. Using obstetrics as an example, the implementation of China’s “three-child” policy in 2021 has led to an increasing proportion of women with advanced maternal age and multiparity. Studies indicate that as maternal age and parity increase, the occurrence of pregnancy complications and adverse pregnancy outcomes also tends to rise, posing new challenges for obstetrics across health care institutions at all levels [2]. Extracting valuable information from obstetric EMRs could significantly benefit clinical research aimed at improving pregnancy success rates.

However, due to varying writing habits among doctors, diagnostic descriptions in medical records lack standardization, which hinders the analysis and use of medical data. Consequently, mapping clinical diagnostic descriptions to a standard terminology database is vital for medical data analysis. This process enables the standardization of medical terms across different health care institutions and regions, preventing misunderstandings and confusion caused by varying terminologies. It positively impacts health care quality, reduces medical costs, enhances doctor-patient relationships, and promotes the development of medical science.

The emergence of large language models (LLMs), represented by ChatGPT, has caused a surge in interest in their application across various fields of research. In the medical domain, LLMs have been extensively used for tasks such as intelligent medical history collection and preliminary diagnosis, personalized treatment and drug recommendations, medical record documentation and report generation, literature retrieval and analysis, and medical education and training [3-6]. Kanjee et al [7] assessed ChatGPT’s ability to accurately diagnose challenging medical cases and suggested that generative artificial intelligence (AI) models hold promise as potential aids to human diagnostic cognition. Research by Agbavor and Liang [8] demonstrated that GPT-3–generated text embeddings can reliably distinguish Alzheimer disease patients from healthy controls and infer cognitive test scores of patients, potentially enhancing early dementia diagnosis. Palanica et al [9] explored ChatGPT’s potential applications in psychological counseling, emotional support, and mental illness screening while discussing related challenges and future research directions.

LLMs have also played a crucial role in medical research. Clinical research often involves large amounts of unlabeled natural language data, and LLMs’ zero-shot learning ability allows them to effectively process such data. Agrawal et al [10] showed that ChatGPT excels in extracting zero-shot and few-shot information from clinical texts. Hu et al [11] revealed ChatGPT’s potential in zero-shot clinical entity recognition tasks. Furthermore, Lamichhane's [12] 3 text-based experiments on mental health classification demonstrated ChatGPT’s potential in zero-shot text classification tasks.

LLMs are chatbot technologies based on natural language processing and deep learning; they learn language patterns and knowledge from a large amount of text data to realize natural conversations with humans. The key to effectively using LLMs is to set an optimal prompt [13].

In few-shot learning, designing appropriate prompts can help LLMs learn better from a small number of training samples and improve performance [13]. Even in zero-shot learning scenarios, appropriate prompts can guide LLMs to use contextual information to output correct results [14]. Prompt engineering has been widely used in various fields of natural language processing, such as question answering, text generation, and sentiment classification, as well as other tasks. By carefully designing prompts, LLMs can better understand the task requirements and context and generate more accurate and useful outputs [13,15,16]. In addition, prompt engineering is an efficient method that does not rely on large-scale computing resources. It can narrow the gap between the pretraining and fine-tuning stages, improve the model’s learning ability and generalization ability on a small amount of data, and fully exploit the model’s potential performance [15].

Chain-of-thought (CoT) prompts were proposed by Wei et al [17], who experimented with the effect of CoT prompts on multiple tasks, including mathematical problems, logical reasoning, reading comprehension, and common sense reasoning; they compared it with other prompt engineering techniques and pointed out that CoT prompts could significantly improve the model’s performance on these tasks and even allow the model to show complex reasoning abilities, such as induction, deduction, and analogy. The basic idea of CoT prompts is that, when giving a question or task, instead of directly asking the model to give an answer or result, the user asks the model to give a CoT, that is, a series of intermediate reasoning steps in which each step is a complete sentence, and the last step is the answer or result. The advantage of this is that it can make the model better understand the meaning and goal of the question or task, avoid irrelevant or wrong outputs, and also make it easier for human users to check and evaluate the model’s output.

The goal of self-consistency prompts is to improve the quality and consistency of the generated results by requiring the model to make consistency judgments on the previously generated text [18]. When using self-consistency prompts, the user first provides an initial text as a prompt and then lets the model continue to generate the subsequent text. Next, the user replaces the “greedy decoding” in the CoT prompt with sampling from the language model’s decoder to generate a set of diverse reasoning paths; finally, the user marginalizes the reasoning paths and aggregates them by selecting the most consistent answer in the final answer. This can force the model to maintain self-consistency when generating text, avoiding contradictions and incoherence.

This paper delves into the potential of LLMs for zero-shot or unsupervised learning in the domain of standardizing diagnostic terminology in obstetrics. By leveraging a composite approach that merges different prompt engineering techniques with LLMs, our goal is to identify the most fitting pipeline for unsupervised scenarios.

As most of the LLMs used in the Chinese domain use the Chinese version of the International Classification of Diseases, 10th revision (ICD-10-CN), as their core training corpus [19], in order to compare the performance of LLMs and supervised learning algorithms horizontally on a baseline, we used standard diagnostic terminology in the ICD-10-CN as the alignment target throughout this study.

## Methods

### Task Overview

**Figure 1:**
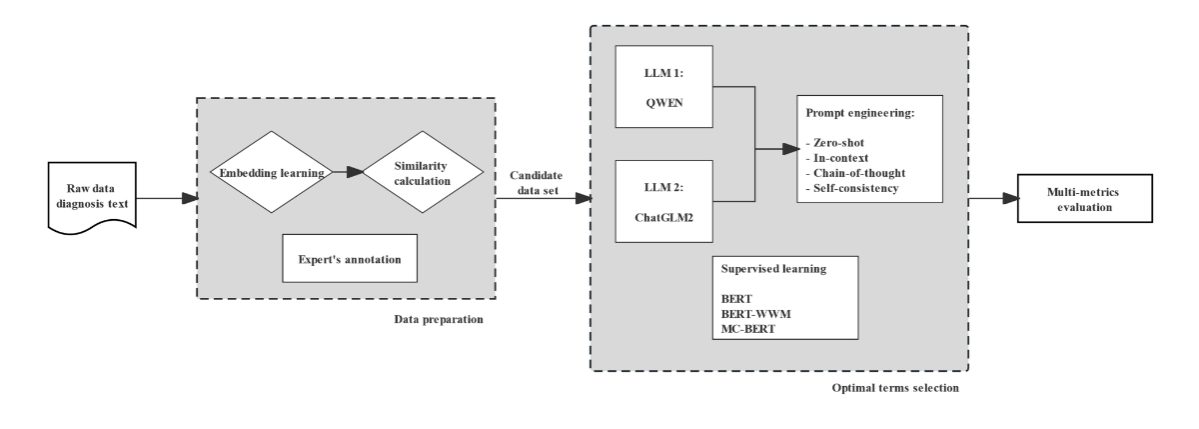
The 4-step approach of this study. For optimal term selection, the combination of a large language model (LLM) and prompt engineering contributes to the unsupervised learning approach to select the optimal terms from 10 candidates. BERT: bidirectional encoder representations from transformers; BERT-WMM: whole word masking BERT; MC-BERT: momentum contrastive learning with BERT; QWEN: Qwen-14B-Chat.

The approach can be divided into 4 steps: (1) mapping the diagnosis in EMRs to the observation domain of the ICD-10-CN via embedded similarity; (2) collecting the candidate mapping terms with similarity above the threshold as the training data set; (3) using 2 LLMs, ChatGLM2 [20] and Qwen-14B-Chat (QWEN) [21], with zero-shot learning to generate the optimal mapping terms; and (4) using 3 pretrained bidirectional encoder representations from transformers (BERTs), BERT [22], whole word masking BERT (BERT-WWM) [23], and momentum contrastive learning with BERT (MC-BERT) [24], for unsupervised generation of the optimal mapping terms for performance comparison. The entire workflow is illustrated in Figure 1.

### Data Preparation

In this study, the raw data were collected from the obstetric EMR data of the People’s Hospital of Guangxi Zhuang Autonomous Region from April 2014 to April 2022; these data contained only diagnostic reports. Sample data are shown in Textbox 1.

**A sample data of diagnoses for ID 720444 is listed below with a translated version. All data processed in this research were in Chinese.**

**Discharge diagnoses**

1. 头位顺产

2. 单胎活产

3. 孕1产1妊娠39+4周

4. 羊水偏少

**Translations**

1. Vertex delivery

2. Singleton live birth

3. Pregnancy: G1P1, 39+4 weeks

4. Oligohydramnios

The raw data set underwent data preprocessing by removing punctuation marks and meaningless special symbols to avoid potential interference with subsequent word segmentation operations.

We implemented LLMs in an intranet security environment. Both ChatGLM2 and QWEN were used exclusively on physically isolated graphical processing units, with access facilitated via OpenAI format and FastAPI (built on PyTorch 2.0). Temperature settings for the LLMs were configured at 0, with *max_token* parameters tailored on a task-by-task basis.

The standard vocabulary referred to in the following text consists of the diagnostic categories belonging to the observation domain of ICD-10-CN.

### Embedding Learning

We used the conditional random fields (CRF) model [24] to segment the text and obtained original-diagnosis raw data aligned with standard vocabulary terms. The principle of CRF is to treat word segmentation as a character position classification problem. Character position information is often defined as follows: *B* represents the beginning of a word, *M* denotes the middle of a word, *E* signifies the end of a word, and *S* indicates a single-character word. Feature functions are constructed to describe the relationship between each character and label and the transition between adjacent labels. Using training data, we learn the weights of feature functions to maximize conditional probability. The Viterbi algorithm predicts new input sequences and finds the most probable label sequence; according to the label sequence, we construct word segmentation results from characters between *B* and *E* and single characters *S*. As shown in Textbox 2, we conducted CRF word segmentation on diagnoses in EMRs.

**Sample of word segmentation with the conditional random fields model. The data below represent a preliminary diagnosis of placenta abruption.**

Original word: 初步 诊 断 为 胎 盘 早剥;

After CRF annotation: 初/B 步/M 诊/M 断/E 为/S 胎/B 盘/M 早/M 剥/E

According to the label, the word segmentation result is as follows: 初步 诊 断/为/胎 盘 早剥.

To calculate the similarity between diagnoses in the raw data set and terms in the standard vocabulary, we used the BERT-medicine model to transform diagnoses and terms into embeddings for storage. The BERT-medicine model is specifically designed to improve the model’s understanding of medical terms and symptoms by introducing a medical domain–specific vocabulary list, lexicon, and pretraining tasks.

The main structure of the BERT-medicine model is the BERT, and the main inputs are the raw word vectors of each word or phrase in the text. In this study, we used diagnoses in the raw data set as the input text sequences. The BERT model extracted the contextual information of the text through a self-attention mechanism and learned the bidirectional linguistic representations, so as to obtain a semantic representation of each word in its context. The final output embedding vector is represented by the sum of character embedding, partition embedding, and position embedding, which constitute the input sequence.

### Similarity Computation

The feature embedding of the diagnosis is denoted by 
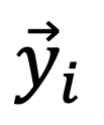
, the feature embedding of the standard terms is denoted by 
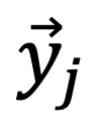
, and their similarity is calculated using the cosine similarity with the following formula:



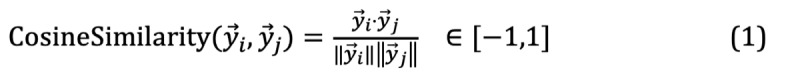



The proposed approach is evaluated through the following steps: Standard terms with a similarity score higher than 0.9 are considered candidates for diagnosis keywords and are then verified by medical experts. The normalized precision and recall are calculated, and the precision-recall curve is obtained. Since a particular diagnosis might have multiple similar standard terms, we aimed to identify as many similar terms as possible, and we thus expected high recall and precision. To obtain candidate terms, we collected the original diagnosis and the 10 most similar standard terms having a similarity score greater than or equal to 0.855.

### Optimal Term Selection

**Figure 2:**
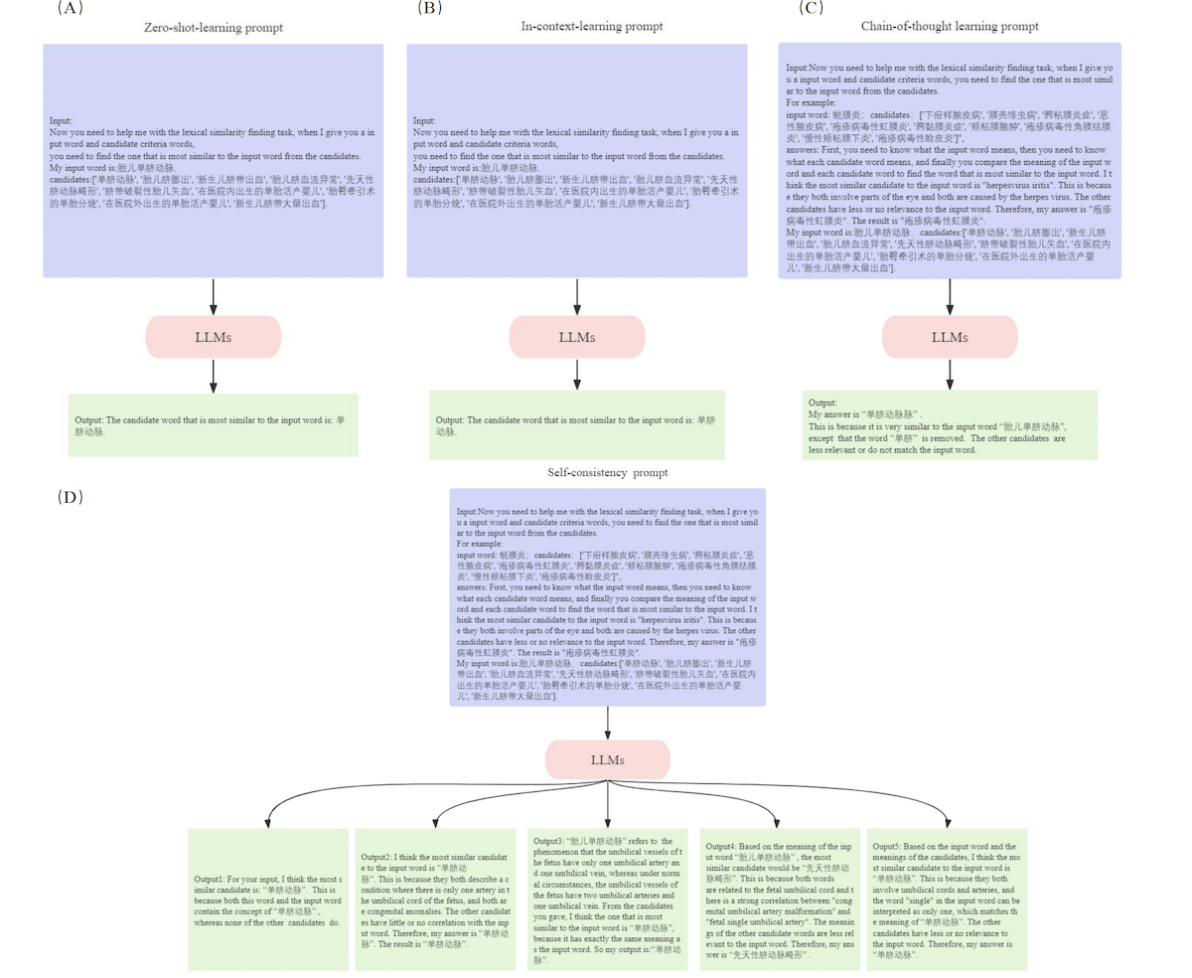
Prompt examples under 4 distinct prompt engineering methods: (A) the zero-shot learning prompt, (B) the in-context learning prompt, (C) the chain-of-thought prompt, and (D) the self-consistency prompt. In the experiment, the raw data being in Chinese led us to use Chinese prompts, which are translated for readability purposes. However, “diagnosis” and “candidate terms” are displayed in their original Chinese format. LLM: large language model.

To comprehensively evaluate the performance of LLMs in the standardization of obstetric diagnostic terminology, we used 4 different prompts, with the prompt design ranging from simple to complex. This started with the prompt trained on zero samples (the zero-shot learning prompt); next were the prompt trained on a small number of samples, the in-context learning prompt, the CoT prompt, and finally the self-consistency prompt. The specific flow chart of LLM training is shown in Figure 2.

The zero-shot learning prompt was meant to guide the LLMs’ output by directly telling them the purpose of this study. The target task of this study was to find the standard-term expression for the diagnosis, that is, to let the LLMs determine the word with the highest similarity. Therefore, we directly told the LLMs to find the most similar word to the input word among the candidate words for the standard term. The LLMs determined the similarity between words based on their own learned knowledge, and then output the word with the highest similarity to the input word as the output result.

The purpose of in-context learning prompts is to give context hints and let LLMs learn by analogy from few shots to output results that more closely meet the requirements [25]. Its input is in the form of {question, answer}, that is, in the input, the question and result are given to the LLM as a template, and it answers the same type of questions in a specific way according to the specific answer.

The input form of CoT prompts is similar to in-context learning prompts, that is, {question, answer}, with the difference that the answer contains the intermediate steps of thinking. In order to reduce human costs, we used LLMs to generate CoT prompts, and then encapsulated them into the prompt inputs.

**Figure 3:**
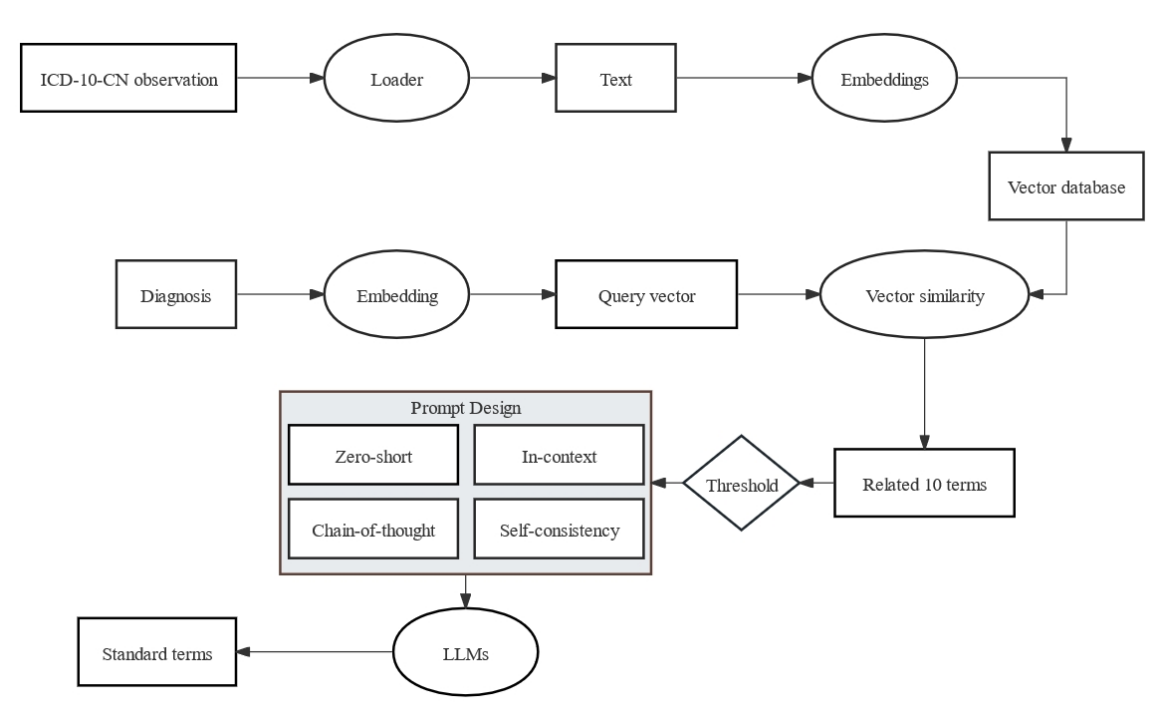
Detailed illustration of the technical intricacies underlying this study. The process of mapping nonstandardized local diagnostic text to standardized International Classification of Diseases, 10th revision, Chinese version (ICD-10-CN) terms involves preliminary similarity-based selection through the vector database, followed by optimal solution selection performed by large language models (LLMs) based on semantic comprehension.

The key method of self-consistency prompts in this study was to input the CoT prompts from the previous section multiple times, obtain multiple results, randomly sample a group of output results, and use the majority voting method to decide the final result. Next, we will demonstrate the experimental process with different prompts through specific examples, shown in Figure 3.

### Evaluation

The evaluation metrics in this study to assess the model’s performance were precision, recall, and *F*_1_-score [26]. We classified words that matched the original word and the standard word as positives, and those that did not match as negatives. There were 4 possible classification outcomes: true positive, in which the model correctly identified a positive as positive; false negative, in which the model mistakenly classified a positive as negative; true negative, in which the model correctly identified a negative as negative; and false positive, in which the model mistakenly classified a negative as positive. Using these classification outcomes, we could calculate precision, recall, and *F*_1_-score to evaluate the model’s performance in standardizing diagnoses.

Precision, recall, and *F*_1_-score (the reconciled mean of precision and recall) were defined as follows:



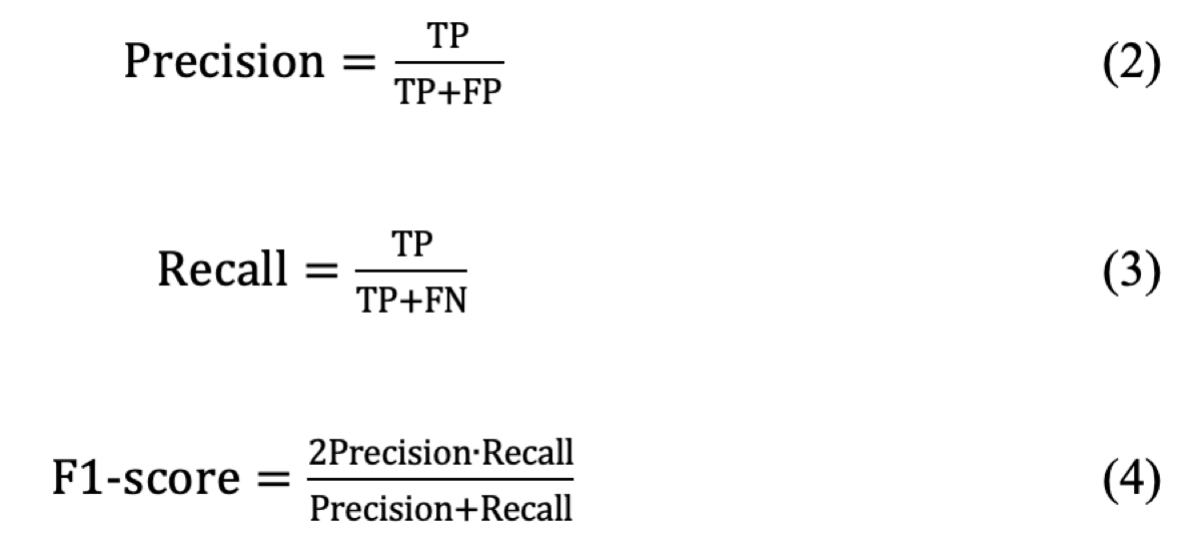



### Ethical Considerations

The study was approved by the People’s Hospital of the Guangxi Zhuang Autonomous Region in China (KT-KJT-2021-67), and all pregnancy data were deidentified and anonymized.

## Results

### Overview

For similarity computation, according to experimental tests, an average precision of 0.88 met the requirement for high precision and recall. The corresponding threshold value at this point was 0.855. Therefore, the threshold value for calculations of similarity was determined to be 0.855, which was used to filter out standard terms that were not similar enough to the diagnosis.

After collecting the candidate data set, we used 2 LLMs and 4 techniques for prompt engineering. Subsequently, we mapped the LLM outputs to the most suitable candidate terms from the ICD-10-CN standard vocabulary, enabling us to calculate precision, recall, and *F*_1_-score. In order to undertake entity normalization, we selected the classic BERT series, comprising BERT, MC-BERT, and BERT-WWM, as our comparison models. We then compared their performance with the results obtained using the LLMs with 4 different prompts. The outcomes of this comparison are presented in Table 1.

**Table 1:** Metric performance comparison across large language model and bidirectional encoder representations from transformers (BERT) series.

Model and prompt engineering approach		Precision, %	Recall, %		*F*_1_-score, %
BERT^a^		91.93	91.95		91.94
Momentum contrastive learning with BERT (MC-BERT)^a^		92.34	92.37		92.35
BERT-whole word masking^a^		92.13	92.17		92.15
**ChatGLM2**					
	Zero shot	75.02		89.90	81.79
**BERT**					
	In context	85.13		86.60	85.85
	Chain of thought	86.52		88.93	82.51
	Self consistency	88.53		90.11	89.31
**Qwen-14B-Chat**					
	Zero shot	84.01		86.72	85.53
	In context	88.25		91.18	89.69
	Chain of thought	89.92		91.30	90.60
	Self consistency	90.91		92.13	91.51

^a^Prompt engineering not applicable to these models.

It is evident from the table that the LLMs and BERT displayed comparable performance at their optimal levels, indicating that the LLMs provided a performance and time advantage under unsupervised conditions. Furthermore, the LLMs exhibited varied performance under different prompt engineering setups. Taking QWEN as an example, the implementation of the self-consistency approach improved the *F*_1_-score by 5% and precision by 7.9% compared to the zero-shot method. Similarly, the same proportion of correctly generated responses was observed in ChatGLM2’s performance, with a range from 9.19% to 18.02%. Thus, QWEN achieved better performance than ChatGLM2 in all 4 prompt engineering approaches.

The BERT series were additional comparison models and exhibited more comparable results in this task. Among the 3 models shown in Table 2, MC-BERT delivered the best performance. However, in this study, the disparity between the 3 versions of BERT was relatively small.

**Table 2:** Cluster results of standardized terms. Original words in Chinese translated to English via ChatGPT.

ID	Word 0	Word 1	Word 2	Word 3	Word 4	Word 5	Word 6
2	α-Thalassemia	β-Thalassemia	δ-β-Thalassemia	Intermedia thalassemia	Major thalassemia	Combined thalassemia	Thalassemia
23	Acute mixed-type fetal distress	Acute fetal distress	Acute fetal heart-type fetal distress	Acute amniotic fluidtype fetal distress	Chronic fetal distress	Chronic fetal -heart type fetal distress	Chronic amniotic fluid–type fetal distress
55	Fetal cardiac malformations	Fetal limb malformations	Fetus with multiple malformations	Fetal ear malformations	Fetal malformations	Fetal structural anomalies	Fetal kidney malformations
73	Uterine interstitial leiomyoma	Uterine subserosal leiomyoma	Uterine intramural leiomyoma	Uterine submucosal leiomyoma	Uterine mucosal leiomyoma	Uterine leiomyoma	Uterine multiple leiomyoma
76	Intrahepatic bile duct stones	Hepatobiliary stones	Biliary stones	—^a^	—	—	—
25	Severe pulmonary arterial hypertension	Mild pulmonary arterial hypertension	Moderate pulmonary arterial hypertension	—	—	—	—
20	Central pelvic stenosis	Pelvic stenosis	Pelvic outlet stenosis	—	—	—	—
36	Acute bronchitis	Acute tracheitis	Chronic bronchitis	—	—	—	—
91	Pregnancy-related reproductive tract infection	Pregnancy-related urinary tract infection	Pregnancy-related urethral infection	—	—	—	—

^a^Not applicable.

### Additional Research

In this study, we used the Louvain algorithm to mine terms from the standard data set output by the LLMs and obtained 1100 relatively common diagnostic terms. In the medical field, different medical institutions and professionals may use different terms to describe the same or similar clinical diagnoses, which can cause difficulties and misunderstandings in data exchange, statistics, and analysis. Therefore, standardizing clinical diagnostic terms is an important task. The standardized terms can be used to unify treatment plans and disease statistics, as well as to build clinical diagnostic knowledge bases. The data in our study were clustered into 107 clusters, and each cluster was analyzed separately, resulting in a diagnostic clustering table. Part of the results of the clustering table are shown in Table 2.

## Discussion

### Principal Results

This paper proposes an effective unsupervised standardization method for obstetric diagnosis. Through a multi-metrics comparison of different LLMs under various prompt engineering strategies, we found that unsupervised LLMs coupled with effective prompt engineering can achieve performance comparable to supervised learning.

A comparison of different prompt engineering strategies showed that although the models’ baseline performance under zero-shot settings varied, they generally showed significant improvement after incorporating strategies such as CoT, which also highlights the importance of effective prompts for LLMs.

The goal of our alignment in this study is the ICD-10-CN terminology, which belongs to the core vocabulary of the Chinese medical field. LLMs trained on Chinese language data usually include it as part of the training corpus [19], and the performance of the baseline model allows prompt engineering to further improve the alignment performance.

### Comparison With Prior Work

Compared to previous research that primarily relied on BERT-based methods to map diagnostic descriptions from EMRs to standard terminologies, this study explores a novel approach based on LLMs. Among BERT models, we identified MC-BERT as the top performer, achieving an *F*_1_-score of 0.9235.

Beyond the conventional BERT methods, we examined 4 mainstream prompt strategies and found that the self-consistency method outperformed the others, achieving an *F*_1_-score of 0.9233. This level of performance matches that of supervised learning, opening up new possibilities for terminology mapping research in the medical domain.

### Limitations

As all data were sourced from real-world patient information, and even though we anonymized the data through multiple strategies and only used a portion of the diagnostic text information without any personal identifying information, there is still a risk associated with uploading patient data to an open network. Additionally, as our research objective was to align and standardize Chinese text based on Chinese target terminologies, the choice of LLMs used in this study was limited. The development of LLMs in the Chinese domain is advancing rapidly, and there are many newly released versions that we have yet to explore.

Moreover, our alignment target was for scientific exploration. In future studies, we will attempt to train target vocabulary that is more suited to the scientific research context into the model through methods such as global optimization and exploring semantic alignment scenarios.

### Conclusions

This paper investigates the capability of LLMs in standardizing clinical medical terms. By using LLMs to standardize diagnostic terms extracted from real-world obstetric EMRs and designing 4 different prompts for LLMs, we were able to compare their output results with those of the BERT model. Our findings demonstrate that QWEN mostly achieved the best performance and had precision on par with the BERT model, which illustrates that an unsupervised approach improved the efficiency of aligning diagnostic terms in daily research and to uncover the hidden value of patient data information.

## Abbreviations

AI: artificial intelligence
BERT: bidirectional encoder representations from transformers
BERT-WMM: whole word masking bidirectional encoder representations from transformers
CoT: chain-of-thought
CRF: conditional random fields
EMR: electronic medical record
ICD-10-CN: Chinese version of the International Classification of Diseases, 10th revision
LLM: large language model
MC-BERT: momentum contrastive learning with bidirectional encoder representations from transformers
QWEN: Qwen-14B-Chat

## Data Availability

The data sets generated during and/or analyzed during this study are not publicly available due to privacy and ethical restrictions but are available from the corresponding author on reasonable request.
